# Artificial intelligence for pediatric fracture detection: impact on diagnostic revisions and patient recall rates in a tertiary emergency setting

**DOI:** 10.1186/s12873-026-01697-3

**Published:** 2026-07-29

**Authors:** Oliver Johannes Deffaa, Johanna Pape, Dominik Schlösser, Franz Wolfgang Hirsch, Martin Lacher, Maciej Rosolowski, Daniel Gräfe

**Affiliations:** 1 Department of Pediatric Surgery, University Hospital, Leipzig, Germany; 2 Department of Pediatric Radiology, University Hospital, Leipzig, Germany; 3 Institute for Medical Informatics, Statistics and Epidemiology, Leipzig University, Leipzig, Germany

**Keywords:** Artificial intelligence, Paediatric radiology, Fracture detection, Diagnostic accuracy, Emergency medicine

## Abstract

**Purpose:**

Artificial intelligence (AI) can support and enhance radiologists in musculoskeletal imaging, but evidence of clinical benefit is still lacking. This prospective study aimed to estimate the range of expected effect size on patient recall rates whether after implementation of an AI system for fracture detection affects in a paediatric emergency setting during out-of-hourse care.

**Methods:**

Children and adolescents (2–18 years) undergoing appendicular skeletal radiography between April and September 2025 during on-call hours at a tertiary referral hospital were eligible. On every second day, automated fracture detection by a commercial AI system (TechCare Kids, Milvue, Paris, France) was available for the treating physician. Endpoints were the rate of diagnostic revisions leading to patient recall the next day, therapeutic changes, length of stay at the emergency department, need for senior consultation, subjective diagnostic confidence, and AI accuracy.

**Results:**

Among 1515 screened patients, 667 were enrolled (median age 11.0 years, 61% male). Fractures were present in 296 cases (44.3%). AI accuracy was 95.1%. Diagnostic revisions occurred in 8.6% without AI, in 5.7% with AI support (risk ratio 0.66; 95% CI 0.37–1.19). Resulting therapeutic changes were rare in both groups (2.0% vs. 0.4%; *p* = 0.10). No significant differences were observed for secondary endpoints, including need for senior consultation (14.3% vs. 12.5%), subjective diagnostic confidence, or length of stay (2.3 h vs. 2.4 h).

**Conclusion:**

Given the small effect size for patient recall rates in an academic medical setting, it is questionable whether the efforts conducting a confirmatory study with adequate statistical power would be justified by the expected incremental benefit for the investigated outcomes.

**Supplementary Information:**

The online version contains supplementary material available at 10.1186/s12873-026-01697-3.

## Key points

AI-based fracture detection achieved high diagnostic accuracy in paediatric emergency radiography.

No significant reduction was observed in recall rates or therapy-altering diagnostic revisions with AI support.

The estimated effect provides a basis for the planning of large confirmatory studies.

**Supplementary Information:**

The online version contains supplementary material available at 10.1186/s12873-026-01697-3.

## Introduction

Over the past decades, the development of deep neuronal networks – particularly convolutional neuronal networks – has led to substantial advances in artificial intelligence (AI)- based analysis [1, 2]. Radiology has played a pioneering role in the clinical adoption of such technologies due to its high degree of digitalisation and the availability of large, annotated datasets. Within musculoskeletal imaging, and especially in the interpretation of conventional radiographs, numerous AI-based software solutions have reached market maturity and are now commercially available [3, 4]. However, only a small proportion of these systems are approved and validated for paediatric use, reflecting the limited availability of suitable training data and the distinct anatomical and developmental characteristics of children [5].

To structure the evaluation of radiology-focused AI systems, van Leeuwen et al. proposed a six-level framework, ranging from technical reproducibility (Level 1) to demonstrated clinical effectiveness and patient outcomes (Level 6) [3]. Most commercially available systems provide evidence only at Levels 1 and 2, typically as part of European Conformity (CE) certification processes [3, 6]. Level 3 studies, which assess the impact of AI on human diagnostic performance, have shown moderate improvements for certain musculoskeletal applications, including in paediatric cohorts [4, 7]. In contrast, evidence at higher levels—particularly studies examining changes in clinical decision-making, therapeutic management, workflow efficiency, or patient outcomes (Levels 4–6)—remains scarce [8]. To date, no robust Level 4 evidence - impact of the software on the patient management decisions - exists in paediatric radiology.

The lack of higher-level evidence is particularly relevant for emergency imaging, where diagnostic accuracy directly influences acute management decisions and delays or errors can lead to patient recall, prolonged recovery, or unnecessary radiation exposure. Early real-world evaluations in paediatric emergency departments have suggested that AI support may improve fracture detection and reduce overlooked injuries [9], but the clinical consequences of such improvements—specifically whether AI reduces diagnostic revisions or alters treatment—have not been systematically assessed. However, the magnitude of the anticipated effect remains uncertain, thereby limiting the feasibility of reliable power calculations for a confirmatory study.

Accordingly, the aim of this exploratory prospective study was to investigate the range of effect size for the influence of an AI-assisted fracture detection system on diagnostic decisions and subsequent therapeutic management in children and adolescents undergoing radiography for suspected traumatic injuries during out-of-hours care in a tertiary referral centre.

## Materials and methods

### Cohort

This prospective, quasi-randomised study included children and adolescents aged 2–18 years who underwent radiographic imaging of the appendicular skeleton during out-of-hours service (16:30–07:30) between April and September 2025. Imaging performed for indications other than suspected fractures, dislocations, or other clinically traumatic bone injuries was excluded. Furthermore, follow-up imaging in patients with known fractures was excluded. All included patients presented to the paediatric surgical emergency department of a tertiary referral hospital and were subsequently referred to the affiliated Department of Paediatric Radiology. Eligibility required the ability to obtain informed consent during the acute visit. Ethical approval was granted by the institutional review board (088/25-ek).

### Clinical setting

The standard institutional workflow was maintained for the study setting: Out-of-hours radiograph interpretation was performed by junior paediatric surgery residents with 3 months to 4 years of clinical experience. All initial diagnostic decisions—including immobilisation, medication, additional imaging, and surgical referral—were made by these emergency physicians. The on-call consultant could be contacted at the physicians’ discretion. Within this standard workflow, all examinations were systematically re-evaluated the following day by at least two board-certified paediatric radiologists during a daily multidisciplinary case conference. Any diagnostic discrepancies were jointly reviewed, and patient recall was initiated when clinically indicated.

### Integration of artificial intelligence

On alternating study days, a commercially available AI tool for automated fracture detection (Milvue Suite, Milvue, Paris, France) was activated. Thus, the presence or absence of AI support followed a predefined every-second-day schedule. The system classified each radiograph into three categories—definite fracture, possible fracture, or no fracture—and displayed the output alongside the original images in real time. Treating physicians retained full autonomy over diagnostic interpretation and management decisions. The AI system was deployed as an on-premises installation and was fully integrated into the clinical workflow. Immediately after image acquisition, radiographs were transmitted simultaneously to the hospitals PACS and the local AI server (without cloud computing). Following automated analysis, AI-generated secondary DICOM images containing the annotation overlays were automatically returned to the PACS and made available for review by the treating physician.

Occasional technical failures of the hospital infrastructure prevented AI availability on approximately 16 study days. These interruptions were unrelated to the AI software itself and were caused by local hardware and integration issues, including hard drive failures, inadvertent server shutdowns, and hospital firewall-related connectivity problems. In accordance with the predefined study protocol, examinations performed during these periods were analysed within the non-AI group.

The reference diagnosis was based on a consensus reading by two senior paediatric radiologists performed the following day, as part of the routine workflow described above, supplemented by additional follow-up imaging when available. Any disagreements were resolved by consensus. This reference standard served as the basis for classifying diagnostic revisions and therapeutic changes.

The AI system was CE-marked and used strictly as a decision support tool. No automated decisions were made without physician oversight, in line with current ethical and regulatory recommendations for clinical AI use.

### Endpoints

The primary endpoint was the occurrence of a diagnostic revision, defined as any discrepancy between the initial emergency department interpretation and the reference standard that required correction (patient recall) the following day. Secondary endpoints included any subsequent relevant therapeutic change, defined as a surgical intervention, the initiation or discontinuation of medication other than analgesics, or a modification of cast or splint immobilisation lasting longer than one week. In addition, the length of stay in the emergency department—from admission to discharge—was recorded for all patients. The frequency with which the on-call senior consultant was contacted, as well as the diagnostic confidence at discharge—rated on a Likert scale from 1 (very low confidence) to 4 (very high confidence)—served as an additional secondary endpoint. Finally, the diagnostic accuracy of the AI system was assessed by comparing its output with the established reference standard.

During the waiting period, patients or their legal guardians completed a four-item questionnaire assessing attitudes toward AI in medical imaging. Each item used a five-point Likert scale (1 = strongly disagree to 5 = strongly disagree). Participation was voluntary and independent of clinical care.

### Statistical analysis

All statistical analyses were performed using R (version 4.3.2; R Foundation for Statistical Computing, Vienna, Austria). Continuous variables are reported as medians with interquartile ranges, whereas categorical variables are presented as absolute and relative frequencies. Group comparisons of categorical variables were conducted using the χ² test or Fisher’s exact test when expected cell counts were fewer than five. Continuous variables that were not normally distributed were compared using the Wilcoxon–Mann–Whitney test. For Likert-scaled outcomes, ordinal logistic regression with cumulative logit modelling was applied. Sensitivity, specificity, positive predictive value (PPV) and negative predictive value (NPV) were calculated.

Risk ratios were calculated using the Katz and Wald methods, respectively, and corresponding 95% confidence intervals were derived using the exact Clopper–Pearson method. A two-sided p-value of less than 0.05 was considered statistically significant. Missing data were assumed to be missing completely at random and were not imputed. All analyses were performed on the predefined population with complete diagnostic information and a known AI status. As a complementary analysis, Bayesian inference was used to quantify the probability that AI assistance reduced the frequency of diagnostic revisions and clinically relevant therapy modifications. Event probabilities in the AI-assisted and control groups were modeled using independent beta-distributed random variables with weakly informative uniform priors (Beta(1,1)). Posterior distributions were obtained for each group and sampled using Monte Carlo simulation (5,000,000 draws). Posterior probabilities of benefit, absolute risk differences, and risk ratios (RRs) were estimated. Uncertainty was summarized using 95% highest posterior density (HPD) credible intervals. To adjust the analyses for age and sex, multivariable Bayesian logistic regression was performed using the stan_glm() function from the rstanarm R package with default weakly informative priors. Uncertainty was summarized using 95% posterior equal-tail percentile intervals for the adjusted log-odds ratios. All Bayesian analyses were considered exploratory and were used to complement the primary frequentist analyses. Logistic regressions were used to adjust the relationship between the AI usage and the outcome (e.g. diagnostic revision) for age and sex.

This study was designed as an exploratory, real-world evaluation of AI-assisted fracture detection in a paediatric emergency setting and was not primarily powered to detect small differences in recall rates.

## Results

### Cohort

During the six-month study period, 1,515 patients met the inclusion criteria. The characteristics were shown in Table 1. Of these, 667 of 1,515 eligible patients (44.0%) provided informed consent and were included in the final analysis. The median age of participants was 11.0 years (IQR 8.1–13.6), and 61% were male. Fractures were identified in 296 of 667 evaluable examinations (44.3%). The diagnostic accuracy of the AI system compared with the reference standard was 95.1%, with an overall sensitivity of 93.0%, specificity of 95.1%, PPV of 92.2% and NPV of 95.6% (Supplementary Material 1).

**Table 1:** Cohort characteristics (age as median with interquartile range)

Screened	1515
Included	667
Age (y)	11,0 (8,1–13,7)
Gender	
- Male - Female	410 (61%) 257 (39%)
Location (% positive for fracture)	
- Hand - Foot - Ankle - Wrist - Elbow - Forearm - Knee - Shoulder - Lower leg - Hip - Upper Arm - Thigh	204 (49%) 108 (38%) 88 (22%) 64 (61%) 55 (49%) 53 (81%) 48 (4%) 18 (61%) 13 (31%) 8 (63%) 6 (100%) 2 (0%)

### Impact on the primary endpoints

The AI system was active on 262 examinations, while 405 examinations were performed without AI support due to the predefined alternating-day schedule and occasional technical failures. When the AI was deactivated, diagnostic revisions were required the following day in 35 of 405 cases (8.6%; 95% CI 6.1–11.8), corresponding to a diagnostic accuracy of the physician of 91.1% (Supplementary Material 2). When the AI system was available, diagnostic revisions occurred in 15 of 262 cases (5.7%; 95% CI 3.2–9.3), yielding a diagnostic accuracy of the physician of 93.9%. The resulting risk ratio for diagnostic revision with AI support versus without was 0.66 (95% CI 0.37–1.19; *p* = 0.24).

Among all diagnostic revisions, nine cases (17.3%) resulted in a relevant therapeutic change. Eight such adjustments occurred on days without AI support, corresponding to 2.0% of all non-AI examinations (95% CI 0.9–3.9%), whereas only one therapeutic change (0.4%; 95% CI 0.01–2.2%) occurred when AI assistance was available (*p* = 0.10). The risk ratio for therapy-altering diagnostic correction associated with AI use was 0.19 (95% CI 0.02–1.53).

**Fig. 1:**
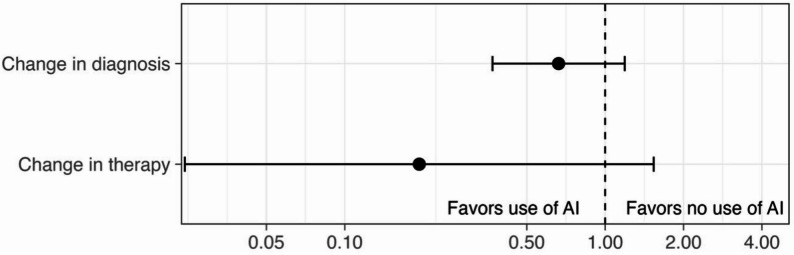
Risk ratio for diagnostic revision or therapeutic change on the following day, depending on whether an artificial intelligence assessment was available

Risk ratio for diagnostic revision or therapeutic change on the following day are presented in Fig. 1.

Bayesian analyses were directionally consistent with the primary frequentist results. Although posterior probabilities favoured a reduction in both diagnostic revisions (86.4%) and clinically relevant therapy modifications (89.0%) with AI assistance, credible intervals remained wide and encompassed the null effect. Analyses adjusted for age and sex led to similar results. Therefore, the data provide only limited evidence for a beneficial effect of AI assistance and do not support a definitive conclusion regarding clinically meaningful outcome improvements. Also in multivariable logistic regression, AI support was not significantly associated with next-day diagnostic changes (β = −0.382, *p* = 0.241). No significant effects of age, sex, or anatomical region were observed.

### Impact on secondary endpoints

Regarding secondary outcomes, treating physicians requested senior consultation in 14.3% of cases without AI support and in 12.5% of cases when AI output was available, a difference that was not statistically significant (*p* = 0.59). Mean subjective diagnostic confidence, assessed on a five-point Likert scale, was identical in both groups (3.6; *p* = 0.91). Similarly, the length of stay in the emergency department did not differ meaningfully between the two conditions, with a median duration of 2.3 h without AI and 2.4 h with AI support (mean difference 0.1 [95% CI − 0.3 to 0.4 h], *p* = 0.75).

### Parental acceptance

**Fig. 2:**
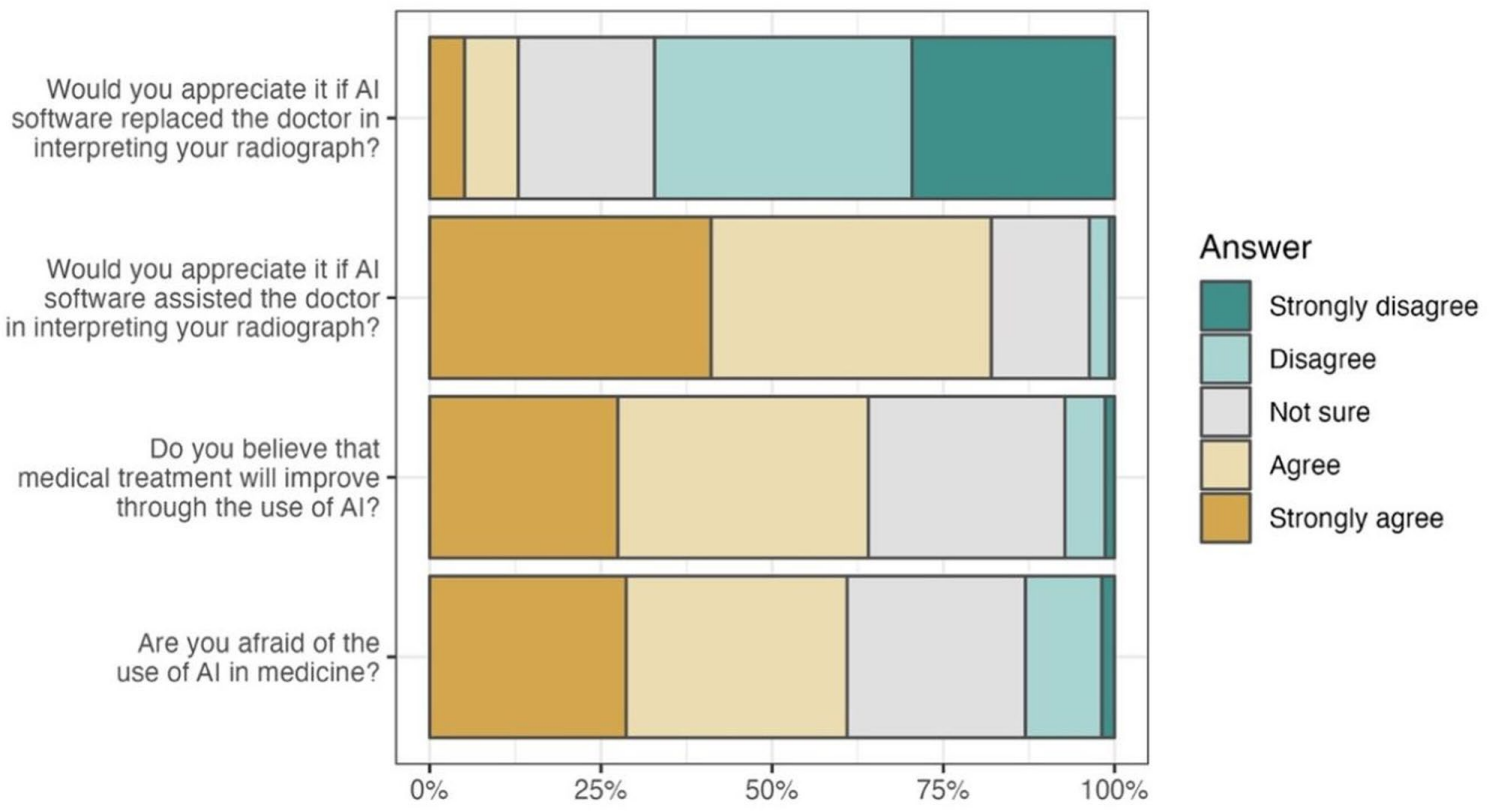
Results of the parent survey regarding acceptance of artificial intelligence support in fracture detection

A total of 650 families completed the questionnaire assessing attitudes towards artificial intelligence in medical imaging. The majority (82%) expressed a positive attitude towards AI when used as a supportive tool for clinicians, whereas only 13% felt that AI systems could replace physicians in the medium term. Sixty-four per cent believed that AI implementation could improve medical care for their children, while 7% disagreed. At the same time, 61% reported feelings of anxiety regarding the use of AI in medicine (Fig. 2).

## Discussion

This prospective study is the first to evaluate the impact of an AI-assisted fracture detection system on diagnostic and therapeutic decision-making in paediatric emergency imaging within a tertiary referral setting. While earlier research has largely focused on technical performance or improvements in reader accuracy, the present work advances the evidence base by examining a higher level of clinical relevance. Specifically, it assesses whether AI support meaningfully reduces diagnostic errors and subsequent patient recall—an outcome with direct implications for patient safety and health service quality.

Activation of the AI system resulted in a lower proportion of diagnostic revisions (5.7% with AI vs. 8.6% without AI), and therapy-altering corrections occurred less frequently when AI support was available. Though the direction and magnitude of the observed effects suggest a potential but modest clinical benefit, descriptive statistics, including Bayesian analysis, does not provide evidence for a beneficial effect of AI assistance and do not support a definitive conclusion regarding clinically meaningful outcome improvements. The limited number of major diagnostic changes in this study likely reflects the highly structured environment of a university hospital, where daily multidisciplinary image reviews and readily available subspecialty expertise contribute to an inherently high baseline diagnostic accuracy. In such settings, opportunities for AI systems to influence clinical decision-making are naturally constrained. Indeed, only nine therapy-modifying corrections occurred over the six-month period, underscoring the rarity of clinically consequential diagnostic errors in this context.

The diagnostic accuracy of the AI tool in this study (95.1%) is consistent with values reported in recent meta-analyses, which demonstrate that AI-based fracture detection in adults achieves accuracies above 90% [10–12]. Although paediatric evidence remains comparatively limited, available studies report similar diagnostic performance [4, 13]. The observed diagnostic accuracy of the AI software (95.1%) confirms the technical maturity of current systems and aligns with international benchmark studies [14], exceeding the accuracy of emergency physicians in this study by 2.6%. Prior research has also shown that AI systems can enhance the performance of non-specialist clinicians [15] and help close the gap between generalists and subspecialists [16]. These benefits appear most pronounced in emergency departments without immediate access to radiology expertise. In contrast, in tertiary referral environments—where junior physicians receive structured training and senior supervision—the measurable added value of AI is inherently constrained.

While an overall accuracy of 95.1% appears high, it is unlikely to be sufficient to support fully autonomous fracture detection. Given the potentially serious clinical consequences of missed fractures or false-positive findings, autonomous diagnostic deployment would require an exceptionally high level of reliability and safety, beyond what is demonstrated by the current performance metrics.

AI support did not influence patient length of stay or the frequency of senior consultations. Reported time savings associated with AI-assisted interpretation are minimal; for example, Kuo et al. documented a mean improvement of only 6.3 s [10]. Such minor gains are unlikely to meaningfully affect workflow efficiency within the broader context of paediatric emergency medicine, where administrative processes and non-imaging clinical tasks dominate overall time expenditure.

Although no significant effects on senior consultation rates or emergency department length of stay were observed in the present study, AI-assisted fracture detection may still offer value through alternative clinical and operational applications. Potential use cases include AI-supported triage, rule-out strategies for low-risk examinations, and workload reduction in settings with limited access to radiology expertise. These outcomes were beyond the scope of the current investigation and may be more relevant in non-academic environments with lower baseline diagnostic performance and fewer specialist resources and a topic of future research.

Parental attitudes toward AI support were overwhelmingly positive. A majority welcomed AI as an assistive tool for clinicians, but only a minority supported full automation—findings consistent with a recent UK national survey [17]. This distinction reflects broader societal expectations that AI should enhance, rather than replace, clinician expertise and underscores the importance of maintaining human oversight in AI-assisted workflows.

This study has several limitations. Unequal group sizes resulted from intermittent server failures, which prevented AI activation on some study days. Potential benefits in terms of radiologists’ workload or reporting times were not evaluated. Recruitment during out-of-hours service posed logistical challenges, and the primary endpoint—diagnostic revision—is rare in high-performing academic settings, further limiting statistical power. Finally, the findings may not be generalisable to non-academic or resource-limited environments, where baseline diagnostic accuracy may differ substantially.

Importantly, the present study was not powered to definitively detect the observed difference in diagnostic revision rates. As indicated by the post hoc power analysis, a substantially larger cohort would have been required to demonstrate statistical significance for an effect of this magnitude. Consequently, the absence of statistical significance should not be interpreted as evidence of no effect, but rather as a limitation in the study’s ability to detect a potentially existing effect.

Future studies should adopt multicentre, randomised designs with larger sample sizes to improve generalisability, particularly in non-university hospitals or emergency departments without dedicated paediatric radiology expertise. Beyond diagnostic revisions and accuracy, future research should incorporate patient-centered metrics such as reattendance rates, radiation exposure from repeat imaging, and health-economic outcomes. Early health technology assessments suggest that AI may reduce unnecessary imaging and prevent costly diagnostic errors [14, 18], highlighting the importance of evaluating cost-effectiveness alongside diagnostic performance.

## Conclusion

In summary, AI-based fracture detection demonstrated high diagnostic accuracy and was well accepted by patients, parents, and clinicians. Yet, in a tertiary referral setting, the measurable benefit by using AI fracture detection for patent recall rates is limited, likely reflecting the already high diagnostic baseline.

## Supplementary Information

Below is the link to the electronic supplementary material.

Supplementary Material 1

Supplementary Material 2

## Data Availability

The datasets generated and analysed during the current study are available from the corresponding author on reasonable request.

## Abbreviations

AI: Artificial Intelligence
CE: Conformité Européenne (European Conformity)
CI: Confidence Interval
IQR: Interquartile Range
