# Supplementary material for: Artificial intelligence for pediatric fracture detection: impact on diagnostic revisions and patient recall rates in a tertiary emergency setting
Source: BMC Emerg Med. 2026 Jul 29;26:204. doi: 10.1186/s12873-026-01697-3 (PMC13421735; doi:10.1186/s12873-026-01697-3)
Supplement: Supplementary file 1 — Supplementary Material 1 [file 12873_2026_1697_MOESM1_ESM.docx]

| **Region** | **Cases** | **Sensitivity** | **Specificity** | **PPV** | **NPV** |
| --- | --- | --- | --- | --- | --- |
| Hip | 8 | 33.3 | 60.0 | 33.3 | 60.0 |
| Elbow | 55 | 91.7 | 100.0 | 100.0 | 93.9 |
| Feet | 108 | 96.9 | 92.1 | 83.8 | 98.6 |
| Hand | 204 | 91.0 | 96.5 | 95.3 | 93.3 |
| Wrist | 64 | 100.0 | 93.1 | 94.6 | 100.0 |
| Knee | 48 | 100.0 | 97.9 | 50.0 | 100.0 |
| Upper arm | 6 | 100.0 | 100.0 | 100.0 | 100.0 |
| Thigh | 2 | NA | 50.0 | 0.0 | 100.0 |
| Shoulder | 18 | 100.0 | 85.7 | 91.7 | 100.0 |
| Ankle | 88 | 64.3 | 95.9 | 75.0 | 93.4 |
| **Overall** | **667** | **93.0** | **95.1** | **92.2** | **95.6** |
| *PPV: positive predictive value; NPV: negative predictive value; NA: non applicable* | | | | | |

Supplemental table 1: Case-wise performance parameters of the artificial intelligence
