# Supplementary material for: Artificial intelligence for pediatric fracture detection: impact on diagnostic revisions and patient recall rates in a tertiary emergency setting
Source: BMC Emerg Med. 2026 Jul 29;26:204. doi: 10.1186/s12873-026-01697-3 (PMC13421735; doi:10.1186/s12873-026-01697-3)
Supplement: Supplementary file 2 — Supplementary Material 2 [file 12873_2026_1697_MOESM2_ESM.docx]

| ID | Sex | Age | AI support | Senior  support | Dx discharge | Dx reference | Region | AI correct | Tx Change | Consequence |
| --- | --- | --- | --- | --- | --- | --- | --- | --- | --- | --- |
| 1 | M | 17.0 | no | no | Fx | None | Elbow | yes |  | No change in the treatment recommendation |
| 2 | f | 9.1 | no | yes | Fx | None | Hand | yes |  | No change in the treatment recommendation |
| 3 | m | 4.8 | no | yes | Fx | None | Foot | yes |  | No change in the treatment recommendation |
| 4 | m | 11.1 | no | yes | Radial fx | Radial fx + Ulna fx | Hand | yes | yes | Surgery |
| 5 | m | 9.1 | yes | no | Radial fx | Radial fx + Ulna fx | Elbow | yes |  | No change in the treatment recommendation |
| 6 | f | 11.7 | no | no | None | Fx | Foot | yes | yes | Buddy taping |
| 7 | f | 8.7 | no | no | None | Fx | Foot | yes | yes | Buddy taping |
| 8 | m | 17.1 | no | no | Fx proximal | Fx | Foot | no |  | No change in the treatment recommendation |
| 9 | f | 11.1 | yes | no | Radial fx | Radial fx + Ulna fx | Lower arm | yes |  | No change in the treatment recommendation |
| 10 | m | 12.9 | no | no | Fx | Additional Fx | Hand | Only 1 fx |  | No change in the treatment recommendation |
| 11 | m | 2.4 | no | no | Fx | None | Foot | yes |  | No change in the treatment recommendation |
| 12 | m | 8.1 | yes | yes | Fx | None | Upper Arm | yes |  | No change in the treatment recommendation |
| 13 | f | 7.8 | no | yes | None | Fx | Ankle | yes |  | No change in the treatment recommendation |
| 14 | f | 9.2 | yes | no | None | Fx | Ankle | yes |  | No change in the treatment recommendation |
| 15 | m | 2.4 | no | no | None | Fx | Hand | no |  | No change in the treatment recommendation |
| 16 | m | 11.4 | no | yes | Fx | Fx | Hand | yes |  | No change in the treatment recommendation |
| 17 | f | 14.6 | no | no | Fx | None | Hand | yes |  | No change in the treatment recommendation |
| 18 | f | 3.7 | yes | no | None | Fx | Hand | yes | yes | Cast |
| 19 | m | 11.9 | yes | no | Fx | None | Hand | yes |  | follow-up after 1 week: asymptomatic --> discontinuation of immobilization |
| 20 | f | 11.2 | no | no | Fx | None | Ankle | no |  | Discontinuation of immobilization after 1 week |
| 21 | f | 11.8 | yes | no | Fx | None | Hand | no |  | No change in the treatment recommendation |
| 22 | m | 6.8 | no | yes | None | Fx | Wrist | yes |  | No change in the treatment recommendation |
| 23 | f | 10.2 | no | no | Fx | None | Foot | no |  | Persistent pain after 6 days, therefore prolonged immobilization |
| 24 | m | 11.4 | no | no | None | Fx | Ankle | yes |  | No change in the treatment recommendation |
| 25 | m | 13.8 | no | no | Fx | None | Hand | yes |  | No change in the treatment recommendation |
| 26 | m | 11.8 | yes | no | None | Fx | Hand | yes |  | No change in the treatment recommendation |
| 27 | m | 13.9 | yes | no | None | Fx | Foot | yes |  | No change in the treatment recommendation |
| 28 | f | 7.7 | no | no | None | Fx | Lower arm | yes | yes | Cast |
| 29 | m | 11.0 | no | no | Fx | None | Hand | yes |  | Discontinuation of immobilization after 1 week |
| 30 | f | 15.3 | no | no | Fx | None | Lower leg | yes |  | No change in the treatment recommendation |
| 31 | m | 2.6 | no | no | None | Fx | Ankle | yes | yes | Cast |
| 32 | m | 5.5 | yes | no | Fx | None | Foot | yes |  | No change in the treatment recommendation |
| 33 | f | 3.3 | yes | no | Fx | None | Ankle | yes |  | No change in the treatment recommendation |
| 34 | m | 16.9 | yes | no | Fx | None | Wrist | no |  | No change in the treatment recommendation |
| 35 | f | 14.0 | no | no | Fx | None | Foot | Old avulusion |  | Discontinuation of immobilization after 1 week |
| 36 | m | 4.4 | yes | no | Fx | None | Hand | yes |  | follow-up after 2 weeks: asymptomatic --> discontinuation of immobilization |
| 37 | f | 4.7 | no | yes | None | Fx | Elbow | yes | yes | Cast |
| 38 | f | 3.0 | no | no | None | Fx | Lower arm | Bowing fracture |  | No change in the treatment recommendation |
| 39 | m | 8.5 | no | yes | Fx | None | Hand | yes |  | follow-up after 1 week: asymptomatic --> discontinuation of immobilization |
| 40 | m | 12.1 | no | no | None | Fx | Foot | yes |  | No change in the treatment recommendation |
| 41 | f | 6.0 | no | no | None | Fx | Lower arm | yes |  | No change in the treatment recommendation |
| 42 | m | 14.2 | no | yes | Fx | None | Hand | yes |  | No change in the treatment recommendation |
| 43 | m | 12.3 | yes | no | Fx | None | Foot | no |  | No change in the treatment recommendation |
| 44 | f | 11.6 | no | no | Fx | Old Fx | Foot | yes |  | No change in the treatment recommendation |
| 45 | m | 4.7 | yes | no | Fx | None | Foot | yes |  | No change in the treatment recommendation |
| 46 | m | 17.7 | no | yes | Fx | None | Ankle | yes |  | No change in the treatment recommendation |
| 47 | m | 6.6 | no | yes | Fx | None | Foot | yes | yes | Cast |
| 48 | f | 16.8 | no | yes | Fx | None | Elbow | yes |  | Recall, immobilization until asymptomatic |
| 49 | f | 8.9 | no | yes | Fx | None | Ankle | Old avulusion |  | No change in the treatment recommendation |
| 50 | m | 10.4 | no | no | None | Fx | Foot | yes | yes | Cast |
| Fx = fracture; Dx = diagnosis; Tx = therapy m = male; f = female; AI = artificial intelligence | | | | | | | | | | |
